# Characterization of Antibiotic Producing Rare Actinomycete *Nonomuraea* sp. JAJ18 Derived from an Indian Coastal Solar Saltern

**DOI:** 10.1155/2014/456070

**Published:** 2014-12-18

**Authors:** Polpass Arul Jose, Kunjukrishnan Kamalakshi Sivakala, Pandiyan Rajeswari, Solomon Robinson David Jebakumar

**Affiliations:** Department of Molecular Microbiology, School of Biotechnology, Madurai Kamaraj University, Madurai 625 021, India

## Abstract

Rare actinomycete genera are accepted as a promising source of novel metabolites having pharmaceutical importance. One such genus of rare actinomycete is *Nonomuraea*. The present study was aimed at characterizing the antibiotic producing *Nonomuraea* strain JAJ18 which was previously isolated from coastal solar saltern. Strain JAJ18 was recognized as a member of genus *Nonomuraea* based on its almost complete 16S rRNA gene sequence and phenotypic characteristics. The strain JAJ18 was found to be closely related to *Nonomuraea maheshkhaliensis* 16-5-14^T^ (98.90%), *Nonomuraea candida* HMC10^T^ (98.58%), and *Nonomuraea jabiensis* A4036^T^ (98.43%). From cell-free culture broth of strain JAJ18, an antibiotic was extracted and purified by silica column chromatography. The obtained antibiotic was found to be active against a range of Gram-positive and Gram-negative bacteria including drug-resistant *Staphylococcus*, with minimal inhibitory concentration (MIC) ranging from 0.5 to 16.0 *µ*g mL^−1^. The structural characteristics of antibiotic were determined by FTIR and NMR spectroscopy. The antibiotic was identified to be an aliphatic rich compound with significant dissimilarity to known antibiotics reported from members of the genus, *Nonomuraea*. As the trends to discover novel metabolites from *Nonomuraea* are vibrant, further studies are needed to understand the structural and biotechnological significance of antibiotic compound produced by *Nonomuraea* sp. JAJ18.

## 1. Introduction

Enduring infectious diseases and rapidly mounting antibiotic resistance have intensified the search for new antibiotics in order to maintain a pool of effective antibiotics against the pathogenic microorganisms. In recent years, rare actinomycetes are considered as potential producers of novel bioactive compounds [1, 2]. The rare actinomycetes that are often very difficult to isolate and cultivate might represent a unique source of novel biologically active compounds [3]. Some genera of this group are* Actinomadura*,* Actinoalloteichus*,* Actinoplanes*,* Amycolatopsis*,* Actinokineospora*,* Acrocarpospora*,* Actinosynnema*,* Catenuloplanes*,* Cryptosporangium*,* Dactylosporangium*,* Kibdelosporangium*,* Kineosporia*,* Kutzneria*,* Microbispora*,* Microtetraspora*,* Nocardia*,* Nonomuraea*,* Planomonospora*,* Planobispora*,* Pseudonocardia*,* Saccharomonospora*,* Saccharopolyspora*,* Saccharothrix*,* Salinispora*,* Streptosporangium*,* Spirilliplanes*,* Thermomonospora*,* Thermobifida*, and* Virgosporangium* [4].

*Nonomuraea* is less known among the rare actinomycete genera as its taxonomic position was revised several times [5]. The genus* Nonomuraea* was originally proposed by Zhang et al. [6] as a member of the family Streptosporangiaceae which forms extensively branched substrate and aerial mycelia. On the basis of detailed polyphasic taxonomical analysis, the genus currently (November 2014) comprises around 36 species and 2 subspecies (http://www.bacterio.net/nonomuraea.html#maheshkhaliensis). The members of this genus have been isolated from various soil and plant samples including mangrove rhizosphere mud [7], cave soil [8], arid soil [9], acidic soil [10], coastal sediments [11], and medicinal plants [12]. Members of genus* Nonomuraea* have been recognized as a valuable source of novel bioactive metabolites showing antimicrobial, anticancer, anthelmintic, and antipsychotic activities. To this date, some antibiotic compounds derived from different species of genus* Nonomuraea* are actinotiocin [13], maduramycin [14], glycopeptide antibiotic A40926 [15], and pyralomicins [16]. The trend to discover novel metabolites from the genus* Nonomuraea* has been augmented, which is a challenging signal for further exploration of this natural compounds producing resource [5].

The* Nonomuraea* sp. JAJ18 taken for this study was previously isolated from a coastal solar saltern [17], which shows antimicrobial activity against a range of bacteria. The objective of this paper was to describe strain JAJ18 to genus level based on its complete 16S rRNA gene sequence and phenotypic characteristics, purify the antibiotic from its culture broth, and disclose structural characteristics and antibacterial potential of the purified compound.

## 2. Materials and Methods

### 2.1. Strains and Their Maintenance

Antagonistic rare actinomycete strain,* Nonomuraea* sp. JAJ18, was previously isolated from a hypersaline coastal solar saltern [17]. Pure culture of this strain was maintained over modified inorganic salt agar slants which contained starch 10.0 g, yeast extract 4.0 g, NaCl 20.0 g, NH_4_SO_4_ 2.0 g, MgSO_4_ *·*7H_2_O 1.0 g, K_2_HPO_4_ 1.0 g, and 22.0 g of agar in 1.0 liter of distilled water. The bacterial test strains, methicillin-resistant* Staphylococcus aureus* (MRSA),* Bacillus subtilis *MTCC 441,* Klebsiella pneumonia* MTCC 109,* Salmonella typhi* MTCC 733, and* Proteus vulgaris* MTCC 426, used in this study for antimicrobial assays were cultured in Mueller Hinton agar slants. The MRSA was obtained from Kovai Medical Center and Hospital, Coimbatore, India. Other bacterial strains were obtained from Microbial Type Culture Collection (MTCC), Institute of Microbial Technology, Chandigarh, India.

### 2.2. Colonial, Cultural, and Physiological Characterization

Cultural characteristics of JAJ18 were observed in twelve different agar media: starch casein agar [20], potato dextrose agar, glycerol nitrate agar [21], asparagine vitamin agar, sucrose nitrate agar, glucose asparagine agar [22], and series of ISP media such as yeast extract maltose agar ISP2, oatmeal agar ISP3, inorganic salt starch agar ISP4, glycerol asparagine agar ISP5, peptone yeast extract iron agar ISP 6, and tyrosine agar ISP 7 [23]. The agar plates were inoculated and incubated at 30°C for 7 d. The plates were then visually examined to determine aerial spore-mass colour, substrate mycelial pigmentation, and the colour of diffusible pigments.

The growth temperature range (15, 20, 25, 30, 37, 45, or 50°C) and pH range (pH 4.5, 5, 6, 6.8, 7.2, 9, and 10) for the growth of strain JAJ18 were determined on modified ISP-4 after culturing for 2 weeks in shake flasks (250 rpm) for 10 days. Salt tolerance of the actinomycete isolate JAJ18 was determined on starch nitrate medium prepared with series of NaCl concentrations between 0 and 30% (0 to 5 M) according to Kushner [24]. Reduction of nitrate, degradation of gelatin and casein, and production of catalase and H_2_S were examined as described by Gordon et al. [25]. Utilization of a range of different sole carbon sources was tested on ISP9 medium [23].

### 2.3. 16S rRNA Gene Amplification and Cloning

For DNA isolation, biomass was obtained by growing the strain JAJ18 in Trypticase soy broth for 7 d at 30°C with agitation in 500 mL flasks containing 100 mL of the medium. From the biomass, DNA was isolated using standard phenol-chloroform extraction procedure [26]. The 16S rRNA gene was amplified from the isolated DNA using universal eubacterial primer set: 27F 5′ AGT TTG ATC CTG GCT CAG 3′ and 1492R 5′ACG GCT ACC TTG TTA CGA CTT 3′ [27]. PCR amplification was carried out in a 50 *μ*L reaction mixture according to Thinesh et al. [28]. The PCR amplified 16S rRNA gene was purified using PCR product purification spin kit (HiPurA, HiMedia, India). Purified 16S rRNA gene amplicon was cloned using pGEM-T Easy vector (Promega) with* E. coli* DH10B cells. The plasmid DNA of the recombinant clone was isolated and sequenced by an automated sequencer (Genetic Analyzer 3130, Applied Biosystems, USA) using the vector-specific primers, SP6 and T7 promoter.

### 2.4. Phylogenetic Analysis

Almost complete 16S rRNA gene sequences of strain JAJ18 and those of other related species derived from GenBank were aligned using ClustalW. Phylogenetic tree was inferred by using suitable programs of the PHYLIP (Phylogeny Inference Package) version 3.68. Nucleotide distances were calculated according to the algorithm of Jukes and Cantor by using DNADIST and a neighbor-joining tree was constructed using NEIGHBOR from the PHYLIP program package [29]. The robustness of tree topology was assessed by bootstrap analysis of the neighbor-joining datasets on 1000 resamplings using the SEQBOOT and CONSENSE options from the same PHYLIP package.

### 2.5. Production and Extraction of Antibiotic

For preparation of seed culture, spore suspensions of JAJ18 were inoculated in modified ISP-4 broth and incubated for 3 d at 28°C on a rotary shaker (220 rpm). A total of 2 mL seed culture was transferred into 200 mL production medium containing starch 10.0 g, yeast extract 4.0 g, NaCl 5.0 g, NH_4_SO_4_ 2.0 g, MgSO_4_ *·*7H_2_O 2.0 g, K_2_HPO_4_ 1.0 g, CaCO_3_ 1.0 g, FeSO_4_ *·*7H_2_O 0.010 g, ZnSO_4_ *·*7H_2_O 0.001 g, MnCl_2_ *·*4H_2_O 0.001 g, and CuSO_4_ *·*5H_2_O 0.001 g in 1 litre of distilled water for antibiotic production. The flask was incubated for 10 d at 30°C on a rotary shaker with 220 rpm. After the incubation period, the culture broth was centrifuged at 10,000 rpm for 10 min to remove the mycelial biomass. Ethyl acetate was added to the supernatants in 1 : 1 proportion and the mixture was agitated for 45 min. The solvent layer was separated and centrifuged at 5000 rpm for 15 min to remove traces of fermentation broth. The ethyl acetate fraction was evaporated and the resultant crude antibiotic was suspended in 50 *μ*L of methanol which was then checked for antibiotic activity.

### 2.6. Purification of Antibiotic

The purification of active fraction was initiated with fractionation of crude extract by silica column chromatography using gradient solvent system (methanol : ethyl acetate gradient 10–100%), monitored by thin layer chromatography (ethyl acetate : methanol, 9.2 : 0.8). The purified fractions were screened for antimicrobial activity by disc diffusion method. Mueller Hinton agar (HiMedia, India) plates were inoculated with the test microorganisms by spreading the microbial inoculums on the surface of the media. Purified fractions were loaded on 6 mm sterile discs, dried, and placed on the surface of the medium inoculated with test microorganism. Plates were incubated at 37°C for 24 h and observed for zone of inhibition around the discs.

### 2.7. Spectral Analysis of Purified Compound

Purified active compound was subjected to partial characterization using a series of spectroscopic methods like FT-IR, NMR, and MS. The FT-IR (KBr) spectrum of the compounds was recorded on Shimadzu FT/IR-8400S spectrophotometer. Band positions are reported in reciprocal centimeters (cm^−1^). Interpretation of FTIR spectrum was done according to infrared frequencies described by Coates [30]. The ^1^H and ^13^C NMR spectra of the compounds in DMSO/CDCl_3_ were measured at 300 MHz and 75 MHz, respectively, on AVANCE 300 NMR spectrometer (Bruker, USA). Tetramethylsilane (TMS) was used as internal standard for calibration of chemical shifts of ^1^H and ^13^C NMR spectra. Chemical shifts were reported in parts per million (*δ*).

### 2.8. Determination of Antimicrobial Potential

Purified compound with antibiotic activity was subjected to determination of minimal inhibitory concentration (MIC) and minimum bactericidal concentration (MBC) to disclose their antimicrobial potential. The MIC values were determined by microdilution broth method as per the guidelines of Clinical and Laboratory Standards Institute [31]. Minimum concentration of compound that showed ≥99.9% reduction of the original bacterial inoculums was determined as the MBC [32]. MIC and MBC values were compared with those of an ideal broad-spectrum antibiotic, erythromycin.

## 3. Results and Discussion

### 3.1. Colonial, Cultural, and Physiological Characteristics of JAJ18

The JAJ18 had pink coloured aerial mycelium and cherry red coloured substrate mycelium without diffusible pigments on modified ISP4. The growth characteristics of* Nonomuraea* sp. JAJ18 on twelve different agar media are summarized in Table 1. The strain JAJ18 grew well on most of the tested agar media except on sucrose nitrate agar, potato dextrose agar, and yeast extract maltose agar. Vegetative mycelium was initially white, yellow, or yellowish red on different media, and it was gradually darkened in old culture. Likewise, aerial mycelia colour was white, mint cream, or wheat colour depending on the medium. Mild diffusible yellow and brick red coloured pigments were observed in ISP2 and ISP3 agar plates, respectively.

The strain JAJ18 was studied for its range of biochemical (Table 2) and physiological (Table 3) characteristics. The strain JAJ18 utilized dextrose, cellobiose, fructose, maltose, rhamnose, salicin, sucrose, trehalose, and sodium citrate. It was negative for hydrolysis of casein and gelatin, growth in Sabouraud dextrose agar and MacConkey agar, and the reduction of nitrate. Growth of JAJ18 occurred in the range of pH (6 to 9) and temperature (25 and 45°C). Good growth was shown in the absence as well as in the presence of NaCl up to 2% (w/v).

Phenotypic properties (growth and biochemical and physiological characteristics) of strain JAJ18 were compared with the nearest valid species* Nonomuraea maheshkhaliensis* 16-5-14^T^,* Nonomuraea candida* HMC10^T^, and* Nonomuraea jabiensis* A4036^T^ (Table 4). Strain JAJ18 differs from its neighbours in its growth and pigmentation on ISP 3 medium, salt tolerance, and carbon source utilization. The JAJ18 produced brick red colour diffusible pigment on ISP3, which has not been observed in closely related strains. In the case of carbon source utilization, disparity was found in its inability to utilize inositol and mannitol.

### 3.2. Molecular Phylogeny of Strain JAJ18

**Figure 1:**
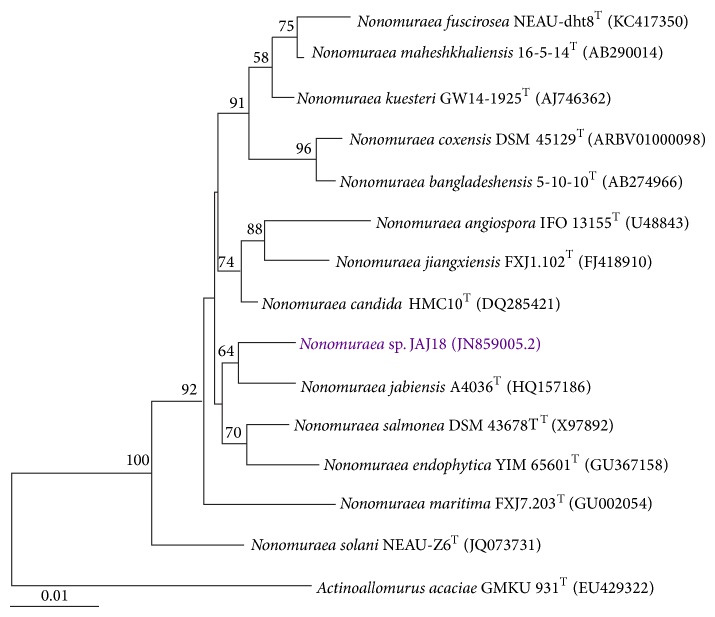
Neighbour-joining tree based on almost complete 16S rRNA gene sequences of JAJ18. The phylogenetic tree shows the relationships between* Nonomuraea* sp. JAJ18 and members of the genus* Nonomuraea*.* Actinoallomurus acacia* GMKU 931^T^ was used as out-group. Numbers at nodes indicate the levels of bootstrap support (%) based on a neighbour-joining analysis of 1000 resampled datasets. Score bar represents 1 nucleotide substitution per 100 nucleotides.

An almost complete 16S rRNA gene sequence of strain JAJ18 was obtained (1484 bp) and submitted to GenBank, with accession number JN859005.2. The 16S rRNA-based phylogenetic analysis showed that strain JAJ18 was closely related to* Nonomuraea maheshkhaliensis* 16-5-14^T^ (98.90%),* Nonomuraea candida* HMC10^T^ (98.58%),* Nonomuraea jabiensis* A4036^T^ (98.43%),* Nonomuraea kuesteri* GW 14-1925^T^ (98.36%), and* Nonomuraea salmonea* DSM 43678^T^ (98.27%). In neighbour-joining phylogenetic tree (Figure 1), strain JAJ18 formed a separate clade with* Nonomuraea jabiensis* A4036^T^.

### 3.3. TLC Profile of Ethyl Acetate Extract from JAJ18

Thin layer chromatograph of ethyl acetate extract of cell-free fermentation broth indicated the presence of five compounds with different Rf values. The chromatograph was developed using iodine vapor and visualized under UV light. The compounds were denoted as 18a, 18b, 18c, 18d, and 18e which had Rf values of 0.067, 0.581, 0.527, 0.5, and 0.45, respectively.

### 3.4. Purification of Antibiotic Produced by* Nonomuraea* sp. JAJ18

An antibiotic produced by* Nonomuraea* sp. JAJ18 was purified using the silica column monitored with TLC. Purification step yielded 13 fractions numbered from J18-1 to J18-13. All the fractions were screened for antibacterial activity against* Bacillus subtilis* by disc diffusion method. Purified fraction J18-04 that showed antibiotic activity was golden yellowish in colour with a greasy appearance. Rf value of the purified fraction was found to be 0.58 in TLC. The purified antibiotic was designated as J18-04.

### 3.5. Spectral Characteristics of Purified Antibiotic, J18-04

Infrared spectra of J18-04 in KBr showed alcohol function (3464.27 cm^−1^), C–H stretch of saturated hydrocarbon (2985.91 and 2937.68 cm^−1^), carbonyl function (1741.78 cm^−1^), conjugated C=O (1654.98 cm^−1^), aliphatic nitro function (1375.29 cm^−1^), C–O bends (1155.40–1047.38 cm^−1^), and =C–H bends (1000–650 cm^−1^). ^1^H-NMR spectrum of antibiotic, J18-04, in DMSO/CDCl_3_ exhibited signals at *δ* H 1.99, 2.15, 2.18, 2.20, 2.50, 5.35, 5.76, 7.24, 7.27, 7.36, 7.38, 7.41, and 7.69. The signals shown in the 0.5-2 and 2-2.5 regions indicate the presence of alkyl protons. Similarly, signals at 5.35 and 5.75 clearly showed presence of alkenes. ^13^C-NMR spectrum of J18-04 exhibited some common main signals at *δ* C 13.76, 18.77, 22.04, 22.40, 28.50, 28.95, 29.06, 31.22, 33.64, and 174.33. The signals between 13.76 and 33.64 indicate carbons of methyl and methylene moieties. The signal at 174.33 indicates the presence of carbonyl carbon.

The spectral data confirmed that purified compound contains more aliphatic units and range of functional moieties such as carbonyl function, aliphatic nitro function, and aliphatic halogen groups. It shared significant dissimilarity with the structural nature antibiotics such as actinotiocin 1500 [13], maduramycin 937 [14], glycopeptide antibiotic A40926 1300 [15], and pyralomicins derived from genus* Nonomuraea*. However, deeper studies will be needed to establish chemical structure of this active compound.

### 3.6. *In Vitro* Antimicrobial Potential of J18-04

The* in vitro* antimicrobial potential of the purified antibiotic from JAJ18 was evaluated against a list of bacteria. The compound J18-04 showed potent inhibitory activity against both of the Gram-positive and Gram-negative bacteria (Table 5). The minimal inhibitory concentrations (MICs) of J18-04 observed for tested bacterial strains were in the range between 0.5 and 16 *μ*g mL^−1^. Similarly, MBC values were in the range between 2 and >16 *μ*g mL^−1^. Moreover, it was found to be comparable with MICs and MBCs of the broad-spectrum antibiotic, erythromycin.

## 4. Conclusion

In summary, the strain JAJ18 was characterized to be a member of rare actinomycete genus* Nonomuraea* and was designated as* Nonomuraea* sp. JAJ18. It is clear that the* Nonomuraea* sp. JAJ18 produces extracellular antibiotic which is effective against a range of bacterial strains including MRSA. The antibiotic purified from* Nonomuraea* sp. JAJ18 was designated as J18-04. Spectral characteristics of purified compound showed presence of a range of functional moieties dissimilar to previously reported antibiotics of* Nonomuraea* species. The results reveal the importance and need of complete structural and* in vitro* pharmacological studies to uncover the industrial importance of identified antibiotic, J18-04.

## Figures and Tables

**Table 1:** Growth characteristics of strain JAJ18.

Agar media	Growth	Substrate mycelium	Aerial mycelium	Pigment
ISP2	Poor	Yellow	None	Golden yellow
ISP3	Good	Brick red	White	Brick red
ISP4	Good	White	White	None
ISP5	Good	Light wheat	Wheat	None
ISP6	Good	Yellowish red	None	Yellowish red
ISP7	Good	Mint cream	Wheat	None
Starch casein agar	Good	Wheat	Dark wheat	None
Potato dextrose agar	Poor	Red	None	None
Glycerol nitrate agar	Good	Light yellow	Mint cream	None
Asparagine vitamin agar	Good	Light yellow	Mint cream	None
Sucrose nitrate agar	Poor	Transparent	Colorless	None
Glucose asparagine agar	Good	Wheat	Light wheat	None

**Table 2:** Biochemical characteristics of strain JAJ18.

Characteristics	Result
Growth on	
Sabouraud dextrose broth	−
MacConkey agar	−
Hydrolysis of	
Casein	−
Gelatin	−
Production of H_2_S	−
Reduction of nitrate	−
Production of catalase	+
Utilization of	
Adonitol	±
Arabinose	±
Cellobiose	+
Dextrose	+
Fructose	+
Inositol	−
Maltose	+
Melibiose	−
Mannose	+
Mannitol	−
Rhamnose	+
Salicin	+
Sucrose	+
Sorbitol	−
Xylose	±
Trehalose	+

+: positive; ±: doubtful/poor; −: negative.

**Table 3:** Physiological characteristics of strain JAJ18.

Characteristics	Result
Growth at initial pH	
5	±
6	++
6.8	++
7.2	++
9	+
Growth at temperature (°C)	
15	−
25	++
37	++
45	+
50	−
Growth in NaCl (%)	
0	++
1	++
2	++
3	±
4	−

++: positive; +: moderate; ±: doubtful/poor; −: negative.

**Table 4:** Comparison of the phenotypic properties of *Nonomuraea* sp. JAJ18 with *Nonomuraea maheshkhaliensis *16-5-14^T^,  *Nonomuraea candida* HMC10^T^, and *Nonomuraea jabiensis* A4036^T^.

Characteristics	JAJ18	HMC10^T^ [18]	16-5-14^T^ [7]	A4036^T^ [19]
Growth on ISP 3 medium				
Aerial mycelium	White	White	White	None
Substrate mycelium	Orange/red	Cream	Light wheat	Brownish orange
Growth on sole carbon sources (1%, w/v)				
Adonitol	±	ND	ND	+
Arabinose	±	±	+	ND
Cellobiose	+	±	+	−
Dextrose	+	ND	+	ND
Fructose	+	+	+	+
Inositol	−	+	±	ND
Maltose	+	ND	ND	+
Mannose	+	+	+	+
Mannitol	−	ND	+	+
Rhamnose	+	+	+	ND
Sucrose	+	+	+	+
Sorbitol	−	ND	ND	−
Xylose	±	+	+	+
Growth temperature range	23–37°C	30–45°C	20–37°C	20–37°C
Growth at 3% NaCl	−	+	+	−

+: positive; ±: doubtful/weak; −: negative; ND: not determined.

**Table 5:** Antimicrobial potential of antibiotic, J18-04, against different bacteria.

Test organisms	MIC (MBC)^*^ (*μ*g mL^−1^)	
	Compound	Erythromycin
*Bacillus subtilis* MTCC 441	0.5 to 2 (>2)	0.5 to 1 (>2)
*Klebsiella pneumoniae* MTCC 109	4 to 8	2 to 3
MRSA (clinical strain)	4 to 16 (>16)	>128 (>134)
*Salmonella typhi* MTCC 733	2 to 3 (>4)	2 to 4 (>4)
*Proteus vulgaris* MTCC 426	2 to 4 (>4)	1 to 4 (>2)

^*^Values in the parenthesis are MBCs.
